# Attention-Based Multi-Scale Convolutional Neural Network (A+MCNN) for Multi-Class Classification in Road Images

**DOI:** 10.3390/s21155137

**Published:** 2021-07-29

**Authors:** Elham Eslami, Hae-Bum Yun

**Affiliations:** Civil, Environmental, and Construction, Engineering Department, University of Central Florida, Orlando, FL 32816, USA; elham.eslami@knights.ucf.edu

**Keywords:** smart infrastructure assessment, road safety, automated pavement condition assessment, convolutional neural network, deep learning

## Abstract

Automated pavement distress recognition is a key step in smart infrastructure assessment. Advances in deep learning and computer vision have improved the automated recognition of pavement distresses in road surface images. This task remains challenging due to the high variation of defects in shapes and sizes, demanding a better incorporation of contextual information into deep networks. In this paper, we show that an attention-based multi-scale convolutional neural network (A+MCNN) improves the automated classification of common distress and non-distress objects in pavement images by (i) encoding contextual information through multi-scale input tiles and (ii) employing a mid-fusion approach with an attention module for heterogeneous image contexts from different input scales. A+MCNN is trained and tested with four distress classes (crack, crack seal, patch, pothole), five non-distress classes (joint, marker, manhole cover, curbing, shoulder), and two pavement classes (asphalt, concrete). A+MCNN is compared with four deep classifiers that are widely used in transportation applications and a generic CNN classifier (as the control model). The results show that A+MCNN consistently outperforms the baselines by 1∼26% on average in terms of the F-score. A comprehensive discussion is also presented regarding how these classifiers perform differently on different road objects, which has been rarely addressed in the existing literature.

## 1. Introduction

According to the 2021 America’s Infrastructure Report Card by the American Society of Civil Engineers (ASCE) [1], road infrastructures in the U.S.A. are graded D on average, showing poor pavement conditions. The current practices of maintenance, repair, rehabilitation, and replacement are not sustainable to restore aging road pavement. State and municipal departments of transportation (DOTs) conduct regular surveys to measure road conditions in terms of (i) cracking and patching, (ii) ride quality, and (iii) rutting. Of these, the first is considered to be the only subjective measure by human inspectors as the other two can be measured accurately using vehicle-mounted accelerators and laser profilers, respectively. To measure cracking and patching, an image-based survey method is often adopted using high-speed line-scanning cameras mounted on a vehicle. The line-scanning cameras can easily collect high-density digital images at a spatial resolution of about 1 mm at a highway speed of higher than 100 km/h. However, many technical challenges still exist regarding the accurate, reliable, and rapid detection, classification, and quantification of various distress and non-distress objects from images collected from large road networks. The challenges are mainly due to (i) variations in image collection conditions, such as camera calibrations, lighting conditions, and image qualities; (ii) variations in the appearance of road distress and non-distress objects in terms of shapes, sizes, orientations, textures, colors, etc.; (iii) the existence of grooving, oil or water stains, dirt or sand, skid marks, leaves, etc.; and (iv) the huge number of images to process for large road networks.

Recent studies have shown remarkable improvements in road image analysis using various computer vision techniques in semi-automated and automated manners [2,3,4,5]. The improvements were possible because of advances in computer hardware and software. On the hardware side, graphics processing unit (GPU)-based parallel processing allows for high-performance computing to process a large amount of image data at a low cost. On the software side, deep learning algorithms, as a data-driven approach, associated with GPUs, have made a significant improvement to the accuracy of road object recognition. Another advantage of deep learning algorithms is that, as a model-free approach, they do not require the explicit representation of the objects to be detected. This is particularly important in the applications of pavement distress recognition as road distresses are highly random in their appearances. Therefore, it is not straightforward to define the features required for traditional model-based approaches. Among the many variations of deep learning algorithms, convolutional neural networks (CNNs) have demonstrated promising results in the applications of pavement distress detection, classification, and segmentation [6,7,8,9].

Although existing studies have shown promising progress on road image analysis, few studies have succeeded in the detection of a wide range of objects using a single image processing algorithm. For an efficient and consistent practice of pavement condition assessment, the development of a single image processing algorithm that can detect various road objects is necessary. The presence of various pavement objects with different sizes and shapes and various surfaces and lighting conditions pose difficulties for deep learning algorithms to classify objects.

**Figure 1:**
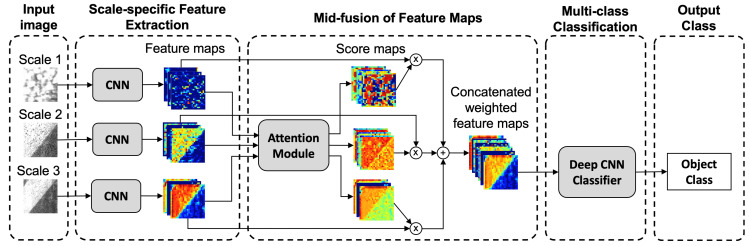
An overview of the presented A+MCNN model. The feature maps are produced by the CNN at each scale and fed to the shared attention module to generate the score maps. The attention module learns to assign scores representing the importance of each feature map. The weighted features are passed to the deep CNN classifier to label the object class.

To address those technical challenges, we present an attention-based multi-scale CNN (A+MCNN) as a novel deep learning algorithm to classify pavement images with 11 different classes, including four distress classes (crack, crack seal, patch, pothole), five non-distress classes (joint, marker, manhole cover, curbing, shoulder), and two pavement classes (asphalt, concrete). The images are collected from both flexible and rigid pavement surfaces using four different high-speed line-scanning cameras to consider variations in camera properties and lighting calibrations. To cope with the variety of pavement objects, we design the A+MCNN to capture contextual information through multi-scale input tiles, as shown in Figure 1. Early fusion, mid-fusion, and late fusion are three approaches that are usually employed to fuse features extracted from multi-scale tiles. We employ an attention module as a mid-fusion strategy to adaptively combine multi-scale features based on their importance for the final prediction.

We present a comprehensive experimental comparison of the state-of-the-art image classifiers, including VGG16, VGG19, ResNet50, DenseNet121, and A+MCNN, in terms of precision, recall, F-score, class separation ability, and computational costs. These extensive comparisons reveal how classifiers perform differently on different pavement objects and how an adaptive attention-based fusion of information improves the classification performance. Furthermore, a parametric study of the A+MCNN is conducted, providing a deep understanding of the effect of architecture choices on the multi-class classification of pavement objects. Our main contributions are summarized as follows:

- We employ the A+MCNN with two unique features that are crucial for improving classification performance: (i) the extraction of multi-scale features using input tiles at three different scales, and (ii) an attention-module for the mid-fusion to produce score maps as weight matrices determining the degree to which feature maps at different scales should contribute to the final class label prediction.
- Using the UCF-PAVE 2017 dataset, the classification task is conducted for a wide range of objects in images collected from two types of pavement in various conditions. Furthermore, quantitative and qualitative comparisons of the state-of-the-art classifiers’ performance on pavement objects are provided.
- The A+MCNN outperforms all compared classifiers by 1∼26% on average in terms of the F-score.

The rest of this paper is organized as follows: Section 2 reviews the previous literature on deep learning-based pavement image analysis as well as employing multi-scale features and attention modules in deep learning algorithms. Section 3 illustrates the architecture of the A+MCNN, and Section 4 describes how the UCF-PAVE 2017 dataset is prepared for experiments. Section 5 shows baseline models, implementation details, and experimental results. Ablation studies including the effect of architectural designs as well as computational costs are provided in Section 6. Finally, Section 7 concludes the paper by summarizing contributions, limitations, and recommendations for future works.

## 2. Related Works

### 2.1. Deep Learning in Pavement Image Analysis

Image-based pavement distress detection has been studied for the past three decades and has traditionally been based on hand-crafted features such as shape and texture [10,11,12,13,14,15]. In the past years, deep learning (DL) methods have been successfully applied to overcome the limitations of the traditional image analysis methods. In deep learning models, features are automatically learned from images at many different levels of abstraction. Therefore, this eliminates the need for human-defined features of distresses, which are often not straightforward for pavement objects due to their highly random appearances. Deep CNNs, a kind of deep learning models, have been applied in various computer vision applications, including image classification [16,17,18,19], image segmentation [20,21,22,23,24], and object detection [25,26,27,28]. Deep CNNs have been also applied to detect different pavement distresses, especially cracks. Those crack detection studies can be divided into three categories: (i) binary or multi-class classification; (ii) semantic (or pixel-level) segmentation; and (iii) object detection.

In binary classification, an image tile, a portion of a pavement image, is classified as a crack or not based on the presence of any crack pixel in the tile. Zhang et al. [29] classified 99 × 99 pixel image tiles, created form 3264 × 2448 pixel pavement images, using a deep CNN. Similarly, Gopalakrishnan et al. [30] used a deep CNN with the transfer learning method to classify pavement images for binary crack classification. They employed a pre-trained VGG-16 on the ImageNet dataset to extract deep features from pavement images before the classifier predicted the labels. For multi-class classification, Li et al. [31] proposed a deep CNN to classify pavement tiles into five categories: longitudinal crack, transverse crack, block crack, alligator crack, and non-crack. They trained deep CNNs with various receptive field sizes using 256 × 256-pixel 3D pavement tiles.

Pixel-wise segmentation assigns a label to each pixel in a pavement image. In the semantic segmentation studies, Zhang et al. [32] proposed CrackNet to segment pavement image pixels into crack or background. CrackNet is a CNN-based model in which hand-crafted features, provided by a feature generator, are fed to two convolution and two fully connected layers. Zhang et al. [33] improved CrackNet’s performance by increasing its learning capability and performance speed in CrackNet II. Comparing to CrackNet, CrackNet II uses a deeper architecture with no hand-crafted features. Zou et al. [34] proposed DeepCrack, in which an encoder–decoder architecture is employed to segment pavement image pixels into crack or non-crack. The encoder extracts crack features while the decoder localizes the cracks in the pavement image. Similarly, Bang et al. [6] applied a deep convolutional encoder–decoder network via transfer learning to segment cracks in black-box images. Lau et al. [35] proposed a U-Net-based [36] network architecture in which the encoder is a pretrained ResNet34 [37] to segment pavement cracks.

In object detection studies [38], objects are localized with a bounding box, and their classes are predicted. Li et al. [39] applied Faster R-CNN [40] to detect cracks and potholes in pavement images. Anand et al. [41] also proposed Crack-pot for real-time crack and pothole detection. The Crack-pot tool is a deep CNN-based model combining texture and spatial features to generate bounding-box candidates and then to predict the object class.

Table 1 provides a summary of the literature reviewed in this section. In the second column, we categorize the methods into two groups: deep learning-based methods (DLs) and non-deep learning-based methods (non-DLs). In the automated detection of road distresses such as cracking, DL is considered to be advantageous over non-DL as DL does not require human-defined features of cracks, which are not always straightforward to define due to the randomness in their appearances. The literature mostly focused on the recognition of a limited set of pavement objects. We believe this gap is due to specific challenges introduced by the high variations in the appearance of distressed and non-distressed objects. Thus, in this study we aim to (1) design a unified image processing algorithm that can classify various pavement objects with high variations in shape and size and (2) provide a deeper insight into the performance and computational cost of current CNN architectures for pavement condition assessment.

### 2.2. Multi-Scale Features in Deep Learning

Multi-scale features that encode contextual information have demonstrated significant improvements in various computer vision applications, such as image classification and segmentation [42,43]. Two approaches have often been employed to exploit contextual information from image data: (i) multi-scale inputs (such as image pyramid) [44,45,46,47] and (ii) multi-scale features, extracted from different layers of a network, through skip connections [24,48,49,50].

In the image pyramid methods, Farabet et al. [22] employed the Laplacian pyramid to generate multi-scale inputs. Multi-scale features are extracted from a shared network, and features from all scales are concatenated to predict pixel-level labels. Eigen and Fergus [44] fed images at three different resolutions sequentially to a deep CNN to generate coarse to fine predictions. Pinheiro and Collobert [51] applied multi-scale images at different stages of a recurrent convolutional neural network (RCNN). Lin et al. [45] resized each input image to three scales and fused the extracted feature maps to generate the unary and pairwise potentials of the conditional random field. Chen et al. [47] used multiple resized input images and merged the extracted multi-scale features using an attention model. It should be noted that our work is different from [47] in that we feed multi-scale image tiles containing various contextual information to the A+MCNN, with the goal of encoding information from neighboring areas for the better classification of a central image tile. Chen et al. [47], on the other hand, resized the same image with different scales (the contextual information was the same across all the scales and simply the size of objects changed).

In the skip connection-based approaches, features are combined in the intermediate layers of CNNs. These features are inherently multi-scale since the receptive field increases throughout the layers. Hariharan et al. [52] defined hypercolumns at a pixel as the concatenation of all features from intermediate layers above that pixel to conduct segmentation and object detection for input images simultaneously. Mostajabi et al. [53] concatenated features that were extracted from zoomed-out regions around a superpixel and fed them to a multi-layer perceptron (MLP) to classify the superpixel. Chen et al. [24] extracted multi-scale features by applying MLP to the input and outputs of pooling layers. A major limitation of these methods is that the training process is not ideal (feature extraction section is usually separated from classifier training) or the training takes a long time.

In road pavement applications, some studies have employed multi-scale features to detect cracks. Komori et al. [54] generated multi-scale images using a Gaussian filter and fused the resulting probability maps based on the Bayesian theorem to detect cracks. Ai et al. [55] developed an SVM-based method to generate the probability of each pixel containing a crack based on multi-scale neighborhood information. Yang et al. [8] exploited context information using a pyramid module in a CNN model for crack detection. Song et al. [7] captured contextual information by using dilated convolution layers to improve crack detection. Sun et al. [56] defined an encoder–decoder architecture with skip connections to combine multi-scale features at various levels for crack segmentation. Konig et al. [57] used a fully convolutional, U-Net based [36] neural network with a pooling function, called the gated scale pooling operation, to merge multi-scale features from different layers of the model. These studies aimed to exploit multi-scale features through skip connections in a CNN model. In other words, multi-scale features are extracted and merged in intermediate layers of CNNs for crack segmentation. Although skip-nets improve segmentation results by exploiting multi-scale features, the training process is not ideal [47]. Furthermore, these studies are limited to applying a multi-scale paradigm for only crack detection.

To the best of our knowledge, no study in the transportation literature has exploited multi-scale feature encoding for multi-class pavement object classification, despite its advantages in classifying objects that are highly random in size and shape as compared to the commonly used single-scale counterparts.

### 2.3. Attention Models in Deep Learning

Attention models have been successfully applied in various deep learning applications, including image classification [58,59,60], object detection [61,62,63], image captioning [64], visual question answering (VQA) [65], machine translation (MT) [66], and action recognition [67]. In image captioning, Xu et al. [64] proposed an attention-based long short-term memory (LSTM) network to caption images by generating one word at every time step related to a spatial region of the image. In machine translation, Luong et al. [68] improved the translation task between German and English in both directions by employing an attention mechanism to align the source word or sentence for each word in the target language sentence. Yang et al. [65] employed attention layers in image question answering, generating high scores for regions in the image that were highly related to the answer. In object detection, Caicedo and Lazebnik [62] used reinforcement learning (RL) with a dynamic attention-action strategy to select the contents that required more attention and transform the bounding boxes accordingly, resulting in a more focused target object. Gregor et al. [59] proposed the Deep Recurrent Attentive Writer (DRAW), employing an attention mechanism to select where to look and write image regions for image generation. Mnih et al. [69] proposed an attention-based recurrent neural network (RNN) that is capable of selecting specific regions in images to be processed at high resolution. The presented attention model is not differentiable, which is necessary for a standard backpropagation during the training. Xiao et al. [58] conducted deep learning-based fine-grained image classification using two attention models: the first attention model was object-level attention to select the most relevant patches for the classification; the second attention model was part-level attention to highlight discriminative parts that differentiated various object classes. To the best of our knowledge, there is no study in the transportation literature that applies the attention module for pavement object classification.

## 3. Method

### 3.1. Network Architecture

**Figure 2:**
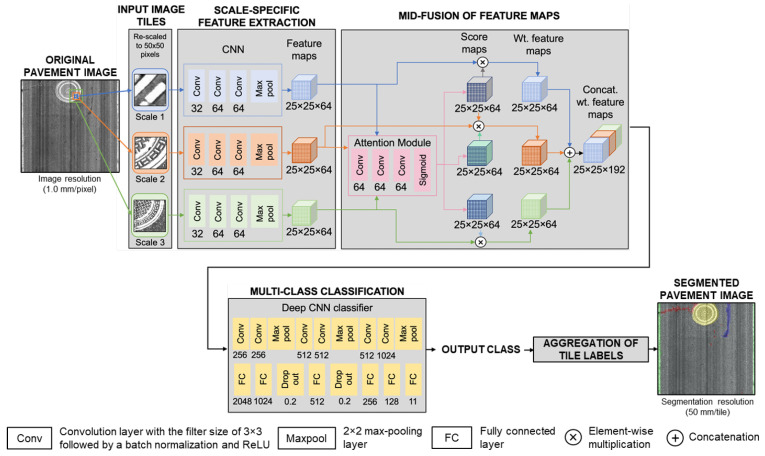
The A+MCNN framework. Input image tiles are created at three scales to encode contextual information. All image tiles are resized to 50×50. Scale-specific features are extracted through separate branches of the CNN for each image tile. Extracted features are passed through the attention module to generate the score maps, reflecting the importance of features at different positions and scales. Weighted features are concatenated to feed the classifier for the final class prediction. The output labels are aggregated to generate the image-level segmentation mask.

The goal of the A+MCNN framework is to classify a pavement image tile into 11 different object classes including four distress classes (crack, crack seal, patch, pothole), five non-distress classes (joint, marker, manhole cover, curbing, shoulder), and two pavement classes (asphalt, concrete). The large and varying sizes of pavement images make it practically difficult or infeasible to feed the full-size images to the deep networks. Thus, we use a patch-wise segmentation strategy with a ≤50-mm spatial resolution that is satisfactory in most road survey applications. To accomplish this goal, the A+MCNN framework consists of five main components as shown in Figure 2: (i) generating input image tiles at three scales; (ii) scale-specific feature extraction; (iii) the mid-fusion of feature maps using an attention module; (iv) multi-class classification; and (v) the aggregation of tile labels to generate the image-level segmentation mask.

The original pavement image in Figure 2 is an 8 bit grayscale image with a 1.0 mm/pixel spatial resolution. This type of image is usually available with commercial line-scanning cameras that are adopted in most state and municipal-level road surveying projects.

Feeding multi-scale input tiles can significantly improve the performance of the A+MCNN compared to single-scale inputs since multi-scale inputs provide the network with more comprehensive contextual information of road objects with various sizes (details in Section 3.2).

In the scale-specific feature extraction step, the CNNs process the input tiles to extract contextual information (i.e., features) at each scale. As a black-box technique, the CNN can extract important features of the image tiles automatically, which is not straightforward to define with traditional hand-crafted feature extraction techniques for road objects with irregular shapes. To obtain more details, each input tile is first passed to the three convolution layers with convolution filter numbers of 32, 64, and 64, respectively. The convolution filter size is 3 × 3 pixels for all three CNNs. Each convolution layer is then followed by a batch normalization layer and a rectified linear unit activation (ReLU), which are not shown in Figure 2 because of space limitations. The CNN produces feature maps $F^{m}$, where *m*∈{1,2,3} is the scale number. A max-pooling layer is applied to the feature maps with a size of 50 × 50 × 64. Applying a 2 × 2 max-pooling layer results in 25 × 25 × 64 feature maps, where 25 × 25 indicates the spatial resolution of feature maps and 64 indicates the number of filters, and each filter represents a visual pattern. It should be noted that, up to this point, the extracted feature maps are processed independently at each scale. In the mid-fusion of the feature maps, the attention module consists of three convolution layers of 1 × 1 × 64, and one sigmoid layer is used to generate the score maps for each scale, $S^{m}$. The size of the score map is 25 × 25 × 64. Then, the weighted feature maps ${\tilde{F}}^{m}$ can be obtained by the inner product as

(1) $${\tilde{F}}^{m}=F^{m}\cdot S^{m}$$

or

(2) $${\tilde{f}}_{w,h,d}^{m}=s_{w,h,d}^{m}\cdot f_{w,h,d}^{m}$$

where *w* and *h* are the x and y-coordinates of the 2D input tile; *d* is the number of convolution filters; ${\tilde{f}}_{w,h,d}^{m}$ is the weighted feature intensity at the spatial position (w,h) for the convolution filter number *d* at the scale *m*; and $s_{w,h,d}^{m}$ is the score (or the weight of contribution to final class label prediction) corresponding to the feature $f_{w,h,d}^{m}$. Then, the weighted feature maps at different scales are concatenated to produce the concatenated weighted feature map with a size of 25 × 25 × 192.

In the mid-fusion of the feature maps, the concatenated weighted feature map is passed to the deep CNN classifier. The classifier consists of a total of six convolution layers and three max-pooling layers. After the sixth convolution layer, no additional convolution process can be added since the size of the processed feature map becomes 3 × 3 × 1024. Then, those six fully connected layers are used to improve the classification performance. Two dropout layers with a rate of 0.2 are used to prevent over-fitting.

In the step of the output class, the class label is determined for the input image tiles by selecting the highest number among the 11 classes specified by the classifier. As the final step, the class determined in the previous step is labeled to the scale-1 region in the original image. Once label predictions for all non-overlapping 50 × 50 tiles corresponding to an image are generated, they are aggregated to create a segmentation mask with the same size and shape as the original image.

In summary, the A+MCNN is improved in two aspects compared with existing deep learning techniques: (i) multi-scale inputs for random-size objects and (ii) an attention module for the mid-fusion of multi-scale feature maps. In the next two subsections, we discuss in detail how these improvements are implemented in this study.

### 3.2. Multi-Scale Inputs

To segment pavement images with an appropriate spatial resolution, the A+MCNN assigns 1 of the 11 classes to a 50 × 50 mm${}^{2}$ region at actual scale, which is equivalent to 50 × 50 pixels in the original image. Although the 50 × 50 mm${}^{2}$ spatial resolution is usually dense enough to localize those distress and non-distress objects, using a 50 × 50 mm${}^{2}$ tile as the input image is not necessarily advantageous in classification.

**Figure 3:**
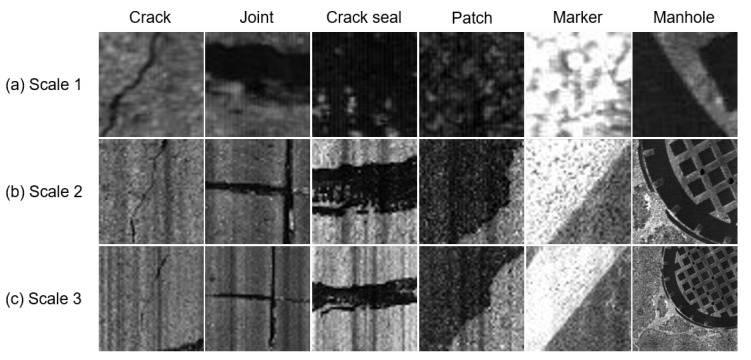
Scope of small, intermediate, and large scales for different objects. (**a**) 50 × 50-mm${}^{2}$ region in scale 1; (**b**) 250 × 250-mm${}^{2}$ region in scale 2; and (**c**) 500 × 500-mm${}^{2}$ region in scale 3.

Figure 3 shows sample image tiles at three scales for different pavement objects. Although a small object, such as a crack, can be easily recognized in the scale-1 image, a large object, such as a patch, is difficult to distinguish from an asphalt background at the same scale due to limited visual scope. On the other hand, the patch can be easily recognized at scale 3, but the crack is difficult to be recognized at scale 3, particularly when the crack is small and/or thin. Scale 2, the intermediate scale between scales 1 and 3, is good to detect the crack seals and the joints that are a middle form of the crack and the patch, but scale 2 is less advantageous than scale 1 for cracks and less useful than scale 3 for patches. Figure 3 illustrates why the multi-scale input tiles are needed for random road objects: (i) the line-scanning camera used in road image collection does not use a zoomable lens, and (ii) the distance between the line-scanning camera and road surface is fixed. Consequently, a single-scale tile with a constant focal length cannot capture enough contextual image information for various objects in pavement condition assessment applications. Therefore, in this study, we use image tiles at scales 1, 2, and 3, which are equivalent to the ROIs of 50 × 50, 250 × 250, and 500 × 500 mm${}^{2}$, respectively. Those tiles are taken from the original image to ensure that the same center position is used. When tiles are taken near the corners and edges, the larger-scale tiles (i.e., scales 2 and 3) can exceed the boundary of the original image. In that case, the exceeded portions of scales 2 and 3 are “zero-padded’’ for the pixel sizes to make up 250 × 250 and 500 × 500 pixels, respectively. Then, the scale-2 and scale-3 tiles are re-scaled to 50 × 50 pixels to obtain the triple input tiles at the same pixel size.

### 3.3. Mid-Level Fusion with an Attention Module

The fusion strategy of the multi-scale CNN (MCNN) features affects the classification performance significantly. In the A+MCNN, we address the feature fusion issues in two aspects: “where” and “how”. For the “where” aspect, three methods can be used for association with classifier models: (i) early fusion, (ii) mid-fusion, and (iii) late fusion. The early fusion approach creates a joint representation of multi-scale input tiles by merging the data at the input level. A shared model is involved in the training pipeline for all three scales, and the final class prediction is generated by that single model. A limitation of this method is that when mixed multi-scale data are presented to the network, the classifier treats all the scales blindly as equal with the same weights. For example, in the early fusion, scales 1 and 3 are treated equally while they cover different regions. In late fusion, each scale is trained in parallel in a separate branch, which provides the network with more flexibility. Then, high-level features are fused at the class-decision level. A limitation of this method is that a separate training process is required to train the weights for different scales, which is not ideal in terms of trainable parameters and computational cost. Furthermore, data fusion at the inference level neglects the inter-relations between scales. Mid-fusion, on the other hand, processes each scale independently until the mid-level features and then merges them and passes them to the rest of the network. However, the information from multiple scales is still treated equally in the fusion.

For the “how” aspect, different feature maps represent different visual patterns. An ideal classification approach should be able to capture the variance in visual patterns and rely on more informative patterns in class prediction. To address this shortcoming, we introduce the attention module to improve mid-fusion as an adaptively weighted aggregation method. After extracting low-level features at three scales, each feature map is weighted through the attention module. The attention module assigns a score between 0 to 1 to the feature maps of each scale in each channel and spatial position. Therefore, each element in the feature map $x_{w,h,d}$ is revised to ${\tilde{x}}_{w,h,d}$, in which scale, channel, and spatial information is considered.

**Figure 4:**
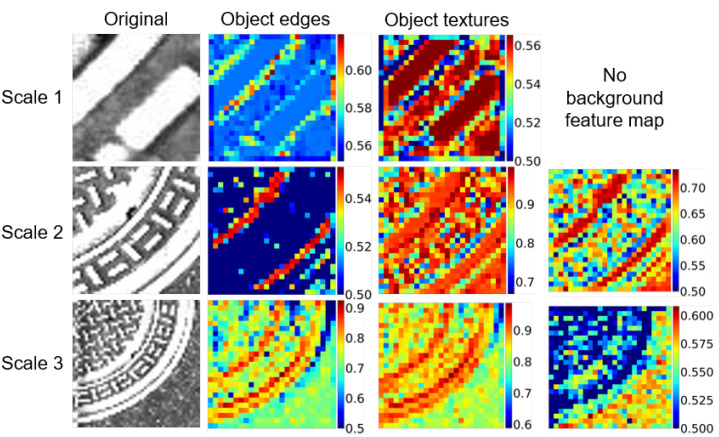
Samples of score maps generated by attention module for manhole tiles at three different scales. While some score maps give higher weights to the object pixels, others highlight object edges or the background.

Figure 4 shows the sample score maps, $s_{w,h,d}^{m}$, of a manhole cover at three different scales. We can see that the attention module localizes the object by giving higher weights to the pixels containing the object class. As we expect, scales 2 and 3 have higher weights (>0.9) for localizing the object comparing to scale 1 (<0.56) with a limited field of view for the manhole. Furthermore, the attention module highlights the edges in some of the feature map channels, helping to discriminate the object. The edges have high scores (>0.8) at scale 3, which is the most informative scale for the manhole cover. The background is also discriminated from the object at scale 3 in some of the feature map channels. As we expect, the weights assigned to highlight the background pixels (<0.6) are lower than the weights generated to highlight the object (>0.9). This helps the network to pay attention to the most informative features for classifying the object.

In summary, we use an attention-based model as a mid-fusion method to generate spatial and channel-wise score maps for features extracted from different input scales. The main purpose of using the attention module in this study is to fuse heterogeneous features of pavement objects that are random in terms of their shape, size, and orientation at different scales. The generated score maps make the network sensitive to unique contextual image features, improving the classification performance for 11 pavement objects. In this way, for example, line-sensitive feature maps at scale 1 will gain higher weights for cracks, while area-sensitive feature maps at scale 3 will gain higher weights for patches.

## 4. Data Preparation for Evaluation

To evaluate the A+MCNN, a series of experiments was performed using the UCF-PAVE 2017 dataset. The dataset included 1215 pavement images, where 719 show asphalt pavement and 496 show concrete pavement. The images were collected with four different high-speed line-scanning cameras with a spatial resolution of 1 mm/pixel. Different camera properties, calibrations, and lighting conditions were represented in the collected dataset.

**Figure 5:**
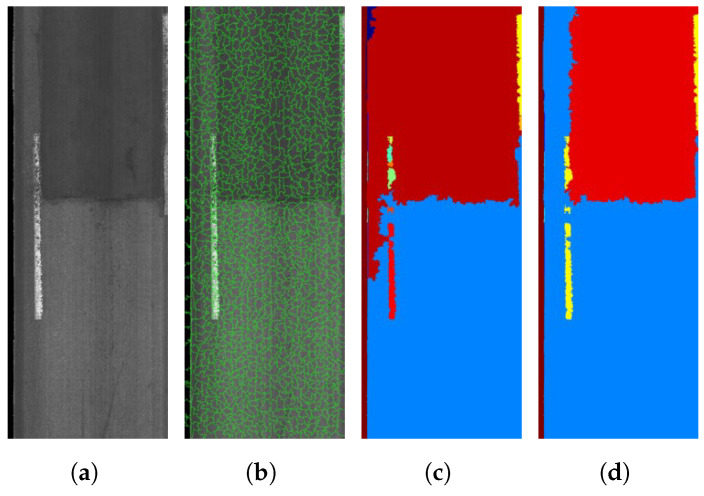
Annotation procedures for areal objects. (**a**) Original image containing an areal object (manhole cover); (**b**) superpixel segmentation; (**c**) unsupervised mean shift clustering to obtain bigger clusters; (**d**) human correction of false clustering and classification.

We developed a software application for semi-automated pixel-level annotation for road objects in two steps: (i) areal object annotation using a superpixel segmentation method [20] and (ii) linear object annotation using the MorphLink-C technique [14]. For areal objects, the Entropy Rate Superpixel Segmentation method [70] was used to divide a road image into small homogeneous clusters, called superpixels, while preserving the edges of objects as shown in Figure 5a,b. Then, an unsupervised mean shift clustering method was used to combine neighboring superpixels into the same class as shown in Figure 5c. For errors in the mean shift clustering, a human annotator could manually correct false clustering and annotate clusters with the correct class as shown in Figure 5d; thus, our annotation approach was semi-automated.

**Figure 6:**
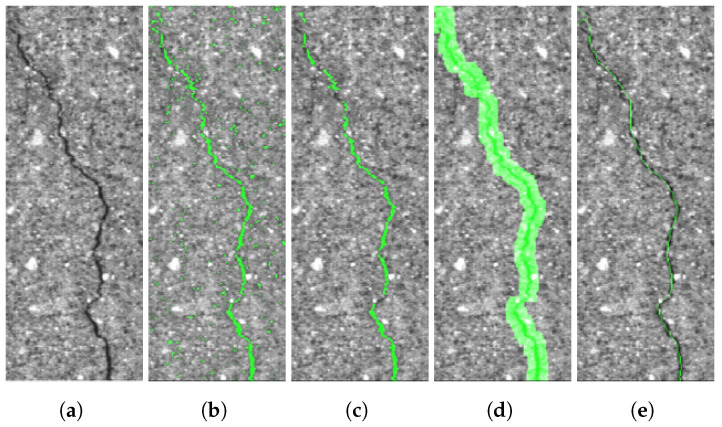
Crack segmentation generated by semi-automated annotation software. (**a**) Original image; (**b**) crack extraction after the bottom-hat transformation; (**c**) area filtering to remove small-pixel noise clusters; (**d**) fragment grouping using the dilation transform; and (**e**) centerline-crack trace using the thinning transformation.

Although superpixel segmentation is effective for areal objects, it is not effective for linear objects, such as cracks and joints. Thus, after the areal object annotation was completed, we annotated linear objects only for background segments (i.e., asphalt and concrete classes) using a series of morphological operations, called the MorphLink. The segmentation procedures for linear objects are shown in Figure 6. After the crack extraction using the bottom-hat operation for dark crack pixels, as shown in Figure 6a,b, we applied the area filter to remove small noise clusters of less than 25 pixels. In Figure 6c, one can notice that the crack pixels exhibit two problems: first, unfiltered noise pixels may still exist since the noise pixel removal is only based on the pixel size without sophisticated characterization of shape, orientation, intensity, etc.; second, the crack in Figure 6c is fragmented into many discontinued crack-pixel clusters. The fragmented crack can be treated as many discontinued small cracks, which can be problematic in the further noise removal process and the characterization of crack properties, such as the number and length of cracks. To label the fragmented crack pixel as a single crack, we applied the dilation transformation with the structuring element of 10 × 10 pixels. Then, the crack pixels within the highlighted area in Figure 6d were considered as a continued single crack. As a semi-automated method, a human annotator could select true continuous cracks for complicated cracks (e.g., block and fatigue cracks) as well as for simple cracks (e.g., single and branched cracks). Using the developed annotation software, we could calculate the averaged crack width according to the total area of the crack pixels in Figure 6d divided by the length of the crack trace in Figure 6e. Determining the crack width is important to measure crack severity in crack surveys and control practices for concrete and asphalt structures.

With the above two-step process, we created a comprehensive image dataset, UCF-PAVE 2017, with pixel-level annotation for four distress objects (crack, crack seal, patch, and pothole), five non-distress objects (joint, marker, manhole cover, curbing, shoulder), and two pavement backgrounds (asphalt, concrete) as shown in Table 2. In UCF-PAVE 2017, we created three-scale input image tiles with the same center position, using the zero-padding method explained in Section 3.2. As the result, the dataset had a total of 10,184,369 input image tiles at each scale, including 7,050,641 from asphalt pavement and 3,133,728 from concrete pavement.

**Figure 7:**
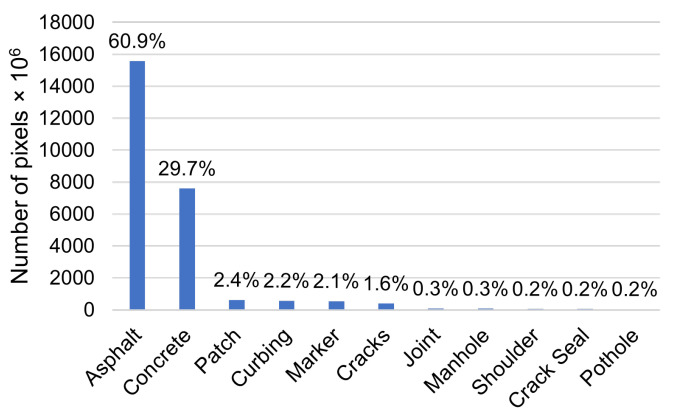
The numbers of pixels for different classes in UCF-PAVE 2017.

The class of input tiles was determined by the following rules: (i) if the asphalt or concrete was the only pixel type in a scale-1 tile, it was categorized into asphalt or concrete, respectively; and (ii) if there were pixels other than the asphalt or concrete, whichever class except for those backgrounds with the largest pixel-count represented the class of the scale-1 tile. Table 2 shows the statistics of scale-1 tile classes in the UCF-PAVE 2017 dataset. Figure 7 shows the numbers of scale-1 pixels for different classes in the dataset. One can see that the dataset was highly imbalanced in terms of class proportions. The background pixels were dominant, occupying 90.6% of the entire dataset. Patch, curbing, marker, and crack pixels accounted for between 2.4% and 1.6% of the dataset. Joint, manhole cover, shoulder, crack seal, and pothole pixels were rare, at less than 0.4% of the entire dataset. An imbalance of the class proportion is usually faced in pavement image analysis. Although the backgrounds were dominant classes in number, the minor classes are more important in pavement condition assessment. Therefore, a robust algorithm needs to be developed for multi-class classification applications. To evaluate the classification algorithms, we used 20% of each class of input tiles for testing, and the remaining 80% for training, from which 20% was kept for validation.

## 5. Experiment Setup and Results

### 5.1. Training

**Figure 8:**
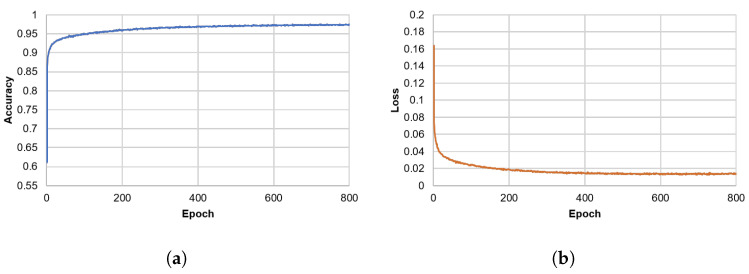
The (**a**) accuracy and (**b**) loss curves for the validation dataset during the training of the A+MCNN. The accuracy and loss continued to improve until becoming saturated.

We trained the A+MCNN in a fully supervised manner. The Adam optimizer with a learning rate of α = 0.0001, $\beta_{1}$ = 0.9, $\beta_{2}$ = 0.999, and ε = 10${}^{-8}$ was used, where $\beta_{1}$ and $\beta_{2}$ are exponential decay rates, and ε is a constant for numerical stability. The network was trained for 800 epochs with a mini batch size of 200. In each epoch, the network used 60,000 random tiles out of more than 6 million tiles in the training dataset. The model with the best performance regarding loss for the validation dataset was selected as the model for use in the testing mode. The training was conducted on an NVIDIA TitanX GPU with a memory configuration of 12 GB. The code was implemented in Python 3.7.3 and TensorFlow 1.14.0. Figure 8 shows the accuracy and loss of validation over 800 epochs during the training of A+MCNN.

### 5.2. Baseline Models for Performance Comparison

**Figure 9:**
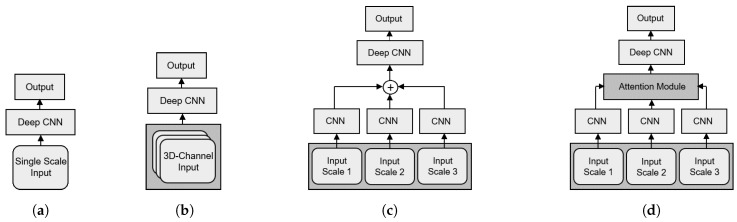
Network structures of (**a**) single-scale CNN, (**b**) multi-scale CNN with early-fusion, (**c**) multi-scale CNN with mid-fusion, and (**d**) attention-based multi-scale CNN (A+MCNN).

To evaluate the performance of the A+MCNN, we compared it with different deep learning classifiers. The classifiers were divided into three categories to understand the effects of different components of the A+MCNN. The first category included the widely used single-scale CNNs, including VGG16, VGG19, ResNet50, and DenseNet121, as shown in Figure 9a. By using the single-scale input of scale 1, the models in this category did not involve the multi-scale fusion process explained in Section 3.2 and Section 3.3. The second category included the CNNs using a 3D channel input image to fuse the three-scale inputs in an early-fusion manner as shown in Figure 9b. M-VGG16, M-VGG19, M-ResNet50, and M-DenseNet121 were the networks in this category used to understand the difference of the early fusion and mid-fusion effects in multi-class classification, where M denotes the models with multi-scale input tiles. For a more direct comparison, MCNN-EarlyFusion was trained with the same trainable layers in the A+MCNN, but it used an early fusion paradigm with no attention module. The third category included the multi-scale CNN model with a mid-fusion strategy and without the attention module (MCNN-MidFusion). The MCNN-MidFusion and the A+MCNN have the same multi-scale inputs and the mid-fusion process, but the MCNN-MidFusion does not have the attention module as shown in Figure 9c. Thus, we were able to understand the effects of the attention module on the classification performance in the comparison of the MCNN-MidFusion and the A+MCNN.

### 5.3. Experiment Results

**Figure 10:**
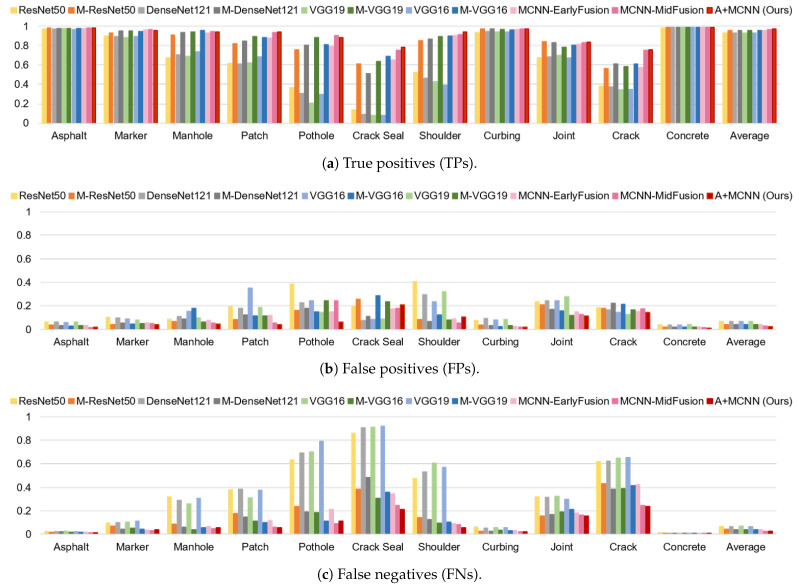
Normalized TP, FP, and FN of each class using different algorithms.

We evaluated the multi-class classification performance of the algorithms in terms of the precision, recall, and F-score:

(3) $$Precision=\frac{TP}{TP+FP},$$

(4) $$Recall=\frac{TP}{TP+FN},$$

(5) $$\text{F-score}=\frac{2TP}{2TP+FP+FN}=\frac{2\times Precision\times Recall}{Precision+Recall}$$

where *TP*, *FP*, and *FN* are true positives, false positives, and false negatives, respectively. The precision determines how many of the positive predictions are truly positive, while the recall shows the ability of the model to predict all relevant instances. The F-score is a harmonic mean of precision and recall and is a useful measure to find the balance between these two metrics. The normalized TPs, FPs, and FNs of each class using different algorithms are shown in Figure 10.

Table 3 summarizes the results of the A+MCNN and the baseline models using the UCF-PAVE 2017 dataset in training, validation, and testing. In Table 3, the last column shows the average performance of each model on all 11 classes, and the best performance of each class is shown in bold. The results show that the A+MCNN outperformed all baseline methods in terms of overall precision, recall, and F-score.

In more detail, we compared the model performances for different classes. Worse performances were observed with the single-scale models (S-CNNs including VGG16, VGG19, ResNet50, DenseNet121) compared to the multi-scale models (M-CNNs including M-VGG16, M-VGG19, M-ResNet50, M-DenseNet121): the average precision was 0.762, the average recall was 0.640, and the average F-score was 0.678 for the single-scale models, while an average precision of 0.883, average recall of 0.855, and average F-score of 0.867 was found for the multi-scale models.

We also observe that the data imbalance affected the classification performance significantly. Joints, manholes, shoulders, crack seals, and potholes were the classes that represented less than 0.4% of each class in the UCF-PAVE 2017 (Group 1), while patches, curbing, markers, and cracks were the classes that made up between 2.4% and 1.6% (Group 2), as shown in Figure 7. The asphalt and concrete classes represented 90.6% of the dataset (Group 3). The analysis results show that the performance increased for the classes with a greater amount of data for all models: the average F-score was 0.720 for Group 1, 0.832 for Group 2, and 0.976 for Group 3. The multi-scale input and the mid-fusion process significantly improved the performance, especially for the classes with a smaller amount of data: for Group 1, the F-score was 0.507 with the S-CNNs, 0.824 with the M-CNNs, 0.840 with the MCNN-EarlyFusion, 0.870 with the MCNN-MidFusion, and 0.889 with the A+MCNN; for Group 2, the F-score was 0.747 with the S-CNNs, 0.864 with the M-CNNs, 0.867 with the MCNN-EarlyFusion, 0.915 with the MCNN-MidFusion, and 0.923 with the A+MCNN; and for Group 3, the F-score was 0.966 with the S-CNNs, 0.979 with the M-CNNs, 0.980 with the MCNN-EarlyFusion, 0.985 with the MCNN-MidFusion, and 0.987 with the A+MCNN. The above results show that the multi-scale input improved the classification performance significantly when the dataset was imbalanced. Among the M-CNNs, the M-VGG16 and M-VGG19 outperformed the M-ResNet50 and M-DenseNet121 slightly for Group 1: the F-score was 0.827 with the M-VGG16 and 0.836 with the M-VGG19, while it was 0.814 with the M-ResNet50 and 0.819 with the M-DenseNet121. The reason for this was that the deeper networks of the M-ResNet50 and M-DenseNet121 need more data to be properly trained. This limitation was mitigated by using the designed networks for Group 1: an F-score of 0.840 was found with the MCNN-EarlyFusion, 0.870 with the MCNN-MidFusion, and 0.889 with the A+MCNN. The results suggest that, with the given data, the customized network design worked better than the state-of-the-art deep networks.

The crack is an important distress class in pavement condition assessment. We can see that the A+MCNN outperformed the VGG16 and VGG19 by 41% for both, and it outperformed the ResNet50 and DenseNet121 by 39% for both, in terms of the F-score. Furthermore, the A+MCNN outperformed the M-VGG16 by 16%, M-VGG19 by 16%, M-ResNet50 by 19%, the M-DenseNet121 by 16%, and the MCNN-EarlyFusion by 13% in terms of the F-score. For the patch, another important distress class, the A+MCNN outperformed the VGG16 by 29%, VGG19 by 28%, ResNet50 by 28%, and DenseNet121 by 28%, in terms of the F-score. For this class, the A+MCNN also outperformed the M-VGG16 by 7%, M-VGG19 by 6%, M-ResNet50 by 9%, M-DenseNet121 by 9%, and the MCNN-EarlyFusion by 7% in terms of the F-score. The above results demonstrate that the A+MCNN improved the classification performance significantly, not only for cracks with small linear shapes but also for patches with large areal shapes. A dramatic performance improvement was observed with the crack seal. The crack seal represents a middle form of the crack and the patch and can be easily misclassified into those two classes. The A+MCNN outperformed the S-CNNs, including improvements on the VGG16 by 82%, VGG19 by 83%, ResNet50 by 74%, and DenseNet121 by 80%. It also outperformed the M-CNNs, including improvements on the M-VGG16 by 11%, M-VGG19 by 14%, M-ResNet50 by 17%, M-DenseNet121 by 20%, and the MCNN-EarlyFusion by 7%.

A pothole can be easily misclassified as a patch, crack, or asphalt because it has pixels with a darker intensity and the existence of cracks around it. Since the A+MCNN calculates appropriate scores for the feature maps at each scale, the pothole can be distinguished accurately from other classes. The A+MCNN significantly outperformed both SCNNs and MCNNs, improving on the VGG16 by 18%, VGG19 by 19%, ResNet50 by 19%, and DenseNet121 by 18%, while improving on M-VGG16 by 6%, M-VGG19 by 2%, M-ResNet50 by 4%, M-DenseNet121 by 3%, and the MCNN-EarlyFusion by 9%.

**Figure 11:**
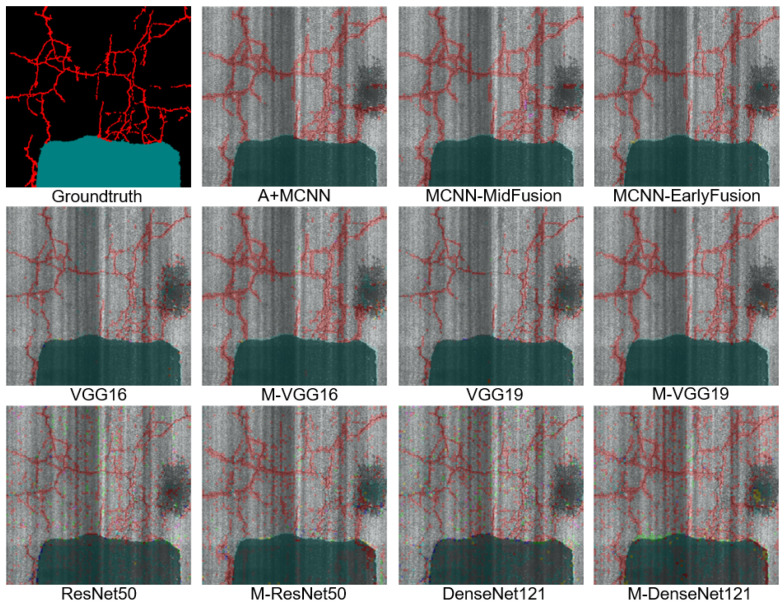
Sample segmentation results of road distresses using different algorithms. Segmentation masks were created by aggregating classification results of 50 × 50 tiles.

Finally, the A+MCNN outperformed MCNN-MidFusion in terms of precision by 2%, recall by 1%, and F-score by 1% on average. On closer inspection of the A+MCNN performance, it is noteworthy that the precision was improved by 14% and F-score by 7% for the pothole class as an important distress in pavement assessment, which had the minimum number of samples in the dataset. This shows the contribution of the attention module in effectively attending to the most discriminative information even in the presence of minimal data while consistently improving upon the results of other classes in terms of the F-score. Figure 11 shows sample segmentation results at the spatial resolution of 50 × 50 mm${}^{2}$ for different algorithms.

In summary, the introduced A+MCNN robustly classified 11 distress and non-distress objects in both asphalt and concrete pavement images with an average of 92% in F-score on entire objects. The A+MCNN outperformed all compared classifiers by consistently improving the classification performance by an average of 1∼26% in terms of the F-score. The comprehensive quantitative and qualitative comparisons in this study, which are barely present in the literature, offer new insights: (1) compared to single-scale CNNs (S-CNNs), the A+MCNN improved the F-score by 24.2% by using multi-scale image tiles to encode the contextual information, and (2) compared to multi-scale CNNs (M-CNNs), the A+MCNN improved the F-score by 4.9% by adapting a mid-fusion strategy with an attention module to assign more importance to the more informative features.

## 6. Discussion

### 6.1. Effects of A+MCNN Parameters

In this section, we evaluate two A+MCNN parameters that could affect the classification performance: (i) the depth of CNNs and (ii) the format of the score maps. First, we conducted experiments with different numbers of CNN layers. As shown in Figure 2, the A+MCNN employs (i) the CNNs for feature extraction with a depth of three layers and (ii) the deep CNN for multi-class classification with a depth of six layers. To understand the effects of the depths of those CNNs, we measured the performances for two cases by doubling the feature-extraction CNNs to 6 layers and by doubling the classification CNN to 12 layers. In the first case, the performance of the A+MCNN decreased by 3.9% in terms of the average precision and 4% in terms of the average F-score, and it increased by 0.5% in terms of average recall. In the second case, the average precision and F-score decreased by 3.8% and 4%, respectively, and the averaged recall increased by 0.9%. This shows the trade-off between the depth of the network and the amount of training data needed. We showed that the originally designed network’s capacity is enough for an optimal training based on our data by adding more layers before or after the attention module, negatively affecting the performance. Since the number of false negatives for distress objects is important in pavement condition assessment, we do not sacrifice the recall to gain higher precision if the overall performance (F-score) does not improve. Therefore, we also investigated the effect of the network depth on the classification of four types of distresses in the dataset: crack, crack seal, pothole, and patch. By increasing the number of convolution layers before the attention module, the performance of the A+MCNN on pavement distresses decreased by 8.2% in terms of the average precision and 8.7% in terms of the average F-score, and it increased by 2.9% for the average recall. By increasing the number of convolution layers after the attention module, the performance of the A+MCNN on pavement distresses decreased by 6.9% for average precision and 6.2% for the average F-score, and it increased by 3.1% for average recall. Therefore, the originally designed network improved the overall performance without unreasonably sacrificing the false negative rate for distress objects.

Second, we compared the performance of the A+MCNN with two different formats of score maps. In Equation (2), the dimensions of the score map for each scale was w×h×d, where *w* and *h* are the x and y-coordinates of the feature map and *d* is the number of convolution filters. The parameters used in Section 5 are *w* = 25, *h* = 25, and *d* = 64. In this way, we applied channel-wise and spatial attention to each scale. To understand the effects of the score map dimension, we compared the performance with the reduced dimension of w×h×1, in which the attention module only generated spatial scores for each scale. In other words, all channels of the feature maps had a shared weight in each scale. The comparison results show that the first setting outperformed the second setting by 1.9% in terms of the average precision, 0.4% in terms of the average recall, and 1.2% in terms of the average F-score. This improvement showed that giving the network the flexibility to give weights to each feature (*d* = 64) improved the performance compared to assigning a similar weight to all the channels corresponding to the same spatial location (*d* = 1). We also compared the performances using the sigmoid and softmax functions to normalize the score maps generated by the attention module. The sigmoid function used in this study increased the average precision, recall, and F-score by 0.3%, 4.4%, and 2.1%. We believe that sigmoid outperformed softmax due to the weighting of the features in each spatial location independently rather than forcing the network to only assign high weights to a single spatial location (softmax). This specifically is important as, for some of the classes in our dataset, the object of interest was distributed over different spatial locations (e.g., crack, crack seal).

### 6.2. Capability of Class Separation

**Figure 12:**
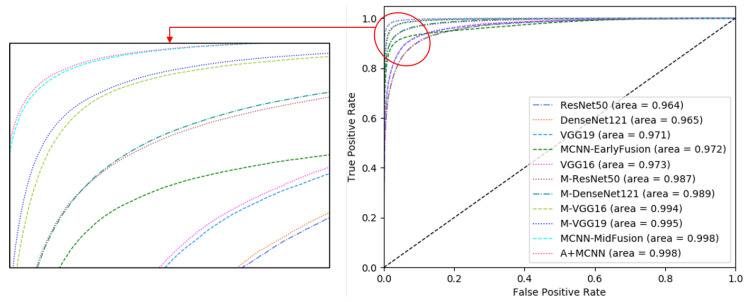
Receiver Operating Characteristic (ROC) curves. Our method achieved the highest area under the curve.

The ability to perform class separation is an important factor that affects the performance of multi-class classification. Figure 12 illustrates the receiver operating characteristic (ROC) curves to compare the performances of the A+MCNN and all baseline methods. The curves summarize the trade-off between the true positive rate and false positive rate for the models. The area under the curve (AUC) indicates the ability of the model to separate the classes. The higher the AUC, the better the model distinguishes the classes. We observe that the MCNN-MidFusion and the A+MCNN have the highest AUCs among the presented models.

**Figure 13:**
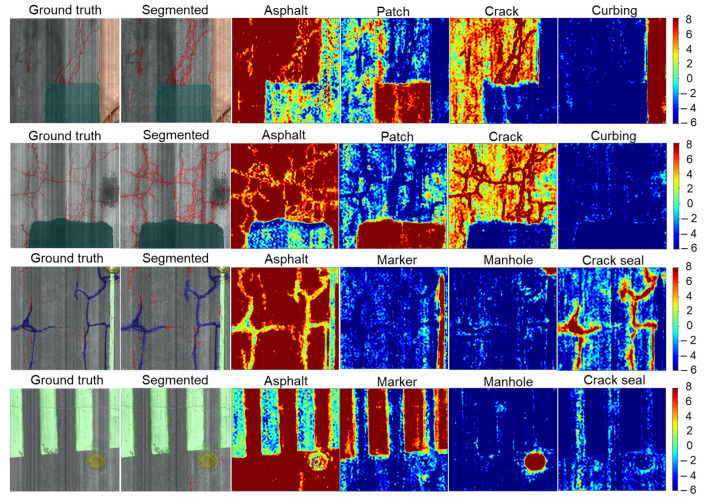
Segmentation results on UCF-PAVE 2017 dataset using the A+MCNN. Hotter colors mean a greater probability that the pixels belong to the specified class.

Figure 13 demonstrates the sample segmentation results generated by the A+MCNN model. Furthermore, the corresponding heatmaps for the pavement classes are plotted for qualitative comparison. A hotter color means a greater probability that the pixels belong to the corresponding class. The heatmaps also show that the A+MCNN model predicts the outputs with a strong class separation.

### 6.3. Computational Costs

Table 4 summarizes the computational costs for different classification approaches used in this study, in terms of the number of trainable variables, training time per epoch, and inference time for 100 batches. While the first column presents the costs for single-scale baselines, including VGG16, VGG19, ResNet50, and DenseNet121, the second column presents the costs for multi-scale baselines including M-VGG16, M-VGG19, M-ResNet50, and M-DenseNet121. Comparing these two columns reveals that the extra computational costs brought by the multi-scale strategy were almost negligible. However, the average F-score increased by 18.9% for multi-scale baselines. Comparing fusion strategies in columns three and four shows that the mid-fusion strategy required more parameters as well as computations but led to a better classification performance (F-score increased by 3.2% compared to the early-fusion approach). Moreover, a fraction of the extra parameters and computations was needed to employ the attention module in A+MCNN to achieve a 1.3% increase in F-score.

### 6.4. Comparison with a Pavement Classifier

In this study, the classification performance of A+MCNN was compared with the state-of-the-art classifiers which are widely used not only in the computer science field but also the transportation literature. Few studies exist addressing the multi-class classification of various distress and non-distress objects simultaneously from both asphalt and concrete pavement images. For a comparison with an existing work on the multi-class classification of pavement objects, we applied the method proposed in [31] to the UCF-PAVE 2017 dataset using both single-scale and multi-scale input tiles. Li et al. [31] introduced a CNN composed of three convolution layers and three fully connected layers to classify pavement image tiles into four different categories of cracks. The classification results are presented in Table 5. The A+MCNN outperformed the CNN in [31] by 39.3% in terms of the F-score. Although using multi-scale input tiles improved the performance of the CNN in [31] by 9.3% in terms of the F-score, it still performed poorly on the classification of objects with a limited number of samples (e.g., pothole and crack seal).

## 7. Conclusions

In this paper, we presented a novel attention-based multi-scale convolutional neural network (A+MCNN) to improve the multi-class classification of asphalt and concrete images in pavement application. This novelty was achieved in two ways: (i) scale-specific features were extracted from multi-scale tiles, covering 50 × 50, 250 × 250 and 500 × 500 pixel regions. Due to the high variation of pavement objects in sizes and shapes, we thus aimed to capture both local and global fields of view for each object; (ii) a mid-fusion strategy combined with an attention module was designed to combine multi-scale features adaptively based on their contribution to classifying a specific pavement object. Weighting original features by these importance factors improves the robustness of classification.

The A+MCNN was evaluated with a comprehensive pixel-level annotated dataset (UCF-PAVE 2017) collected by four different line-scanning cameras. The ground-truth dataset included a total of 1215 annotated asphalt and concrete pavement images with 11 distress and non-distress classes. The A+MCNN outperformed all compared CNN classifiers (VGG16, VGG19, ResNet50, DenseNet121, and the generic CNN). We also investigated the effect of encoding multi-scale contextual information, fusion strategies, and the proposed attention module on the classification of each pavement object.

Our experiments showed that a multi-scale paradigm in the A+MCNN significantly improved the classification performance by 24.2% in terms of the F-score compared to a single-scale approach. The mid-fusion strategy used for combining multi-scale features in the A+MCNN further improved the classification performance by 4.9% in terms of the F-score on average compared to the early-fusion of multi-scale input tiles. Employing the attention module provided additional benefits. For example, the classification performance was improved with the attention module as much as 7% in terms of the F-score for the pothole class, which had the least number of images in the training data (only 0.2% of the training data). When a network does not have a large number of samples during the training, it is extremely important to attend to the most informative parts (scales in our case) of the data to learn the most information possible. This is exactly where the attention module is beneficial to obtain a consistent improvement of classification performance for all classes. The extra computation times incurred by the multi-scale paradigm, mid-fusion strategy, and attention module for the inference mode were 5%, 45.5%, and 8.7% on average.

The parametric study of the A+MCNN provided deeper insights into the effect of the network depth and the attention mechanism. Due to the trade-off between the depth of the network and the complexity of the dataset, doubling the depth of the A+MCNN in scale-specific feature-extraction layers and the deep classification layers decreased the performance by 3.8% and 4% in terms of the F-score. Therefore, deeper networks improve the object classification performance for pavement images only to a certain extent. Our investigation also showed that applying channel-wise and spatial attention to each scale is more beneficial (F-score improved by 1.2%) than spatial attention.

This study makes a four-fold contribution. First, we show that encoding contextual information, especially while dealing with pavement objects with high variations in shape and size, significantly improves the classifiers’ performance. Second, we show that investigating information fusion strategies and showing a mid-fusion strategy is the most impactful strategy. Third, we propose an attention-based mid-fusion strategy to adaptively weight the features to increase robustness and improve the performance even more. Fourth, we study and provide deeper insights into the performance of different architectures, network depths, and computational costs of pavement object classification.

One limitation of this study is that our approach provides a patch-level segmentation mask for pavement images. Although the 50 × 50 mm${}^{2}$ spatial resolution used in this study is acceptable in most road surveys, a pixel-level segmentation mask is required for some pavement applications, such as crack width measurements. Therefore, one research direction could be the semantic segmentation of images for various pavement objects. Furthermore, employing unsupervised learning techniques for image-based pavement analysis, removing the need for annotated training data, is another research direction that could be followed in future studies. 

## Figures and Tables

**Table 1:** Image processing algorithms for pavement object recognition.

References	Methods	Tasks	Objects	Pavement Types
[10]	Local Binary Pattern (Non-DL)	Segmentation	Crack Only	Asphalt
[11,14]	MorphLink-C (Non-DL)	Segmentation	Crack Only	Asphalt
[12]	Wavelet Transform (Non-DL)	Segmentation	Crack Only	Not Specified
[32,33]	CNN (DL)	Segmentation	Crack Only	Asphalt
[34,35]	CNN (DL)	Segmentation	Crack Only	Not Specified
[15]	Superpixel (Non-DL)	Segmentation	Marker, Patch, Manhole, Crack seal	Asphalt
[29]	CNN (DL)	Classification	Crack Only	Not Specified
[30]	CNN (DL)	Classification	Crack Only	Asphalt+Concrete
[31]	CNN (DL)	Classification	Longitudinal Crack, Traverse Crack, Block Crack, Alligator Crack	Asphalt
[13]	Shape & Texture Features (Non-DL)	Detection	Pothole Only	Asphalt
[38]	CNN (DL)	Detection	Lateral Crack, Longitudinal Crack, Alligator Crack, Pothole, Well Cover	Asphalt
[39]	CNN (DL)	Detection	Crack, Pothole	Asphalt
[41]	CNN (DL)	Detection	Longitudinal Crack, Transverse Crack, Patch, Pothole	Asphalt

**Table 2:** UCF-PAVE 2017 database.

Types	Labels	Asphalt Pavement			Concrete Pavement			All Pavement		
		Number of Tiles	Avg. Number of Tiles/img	Std. Number of Tiles/img	Number of Tiles	Avg. Number of Tiles/img	Std. Number of Tiles/img	Total Number of Tiles	Avg. Number of Tiles/img	Std. Number of Tiles/img
Distress objects	Crack (CRK)	383,637 (5.4%)	533	723	73,325 (2.3%)	147	288	456,962 (4.5%)	376	616
	Crack seal (CRS)	18,797 (0.3%)	26	213	4812 (0.2%)	9	41	23,609 (0.2%)	19	166
	Patch (PAT)	229,021 (3.2%)	318	960	33,955 (1.1%)	68	246	262,976 (2.6%)	216	765
	Pothole (POT)	16,912 (0.2%)	23	208	11 (0.0%)	0	0	16,923 (0.2%)	13	160
Non-distress objects	Joint (JNT)	0 (0.0%)	0	0	96,684 (3.1%)	194	147	96,684 (0.9%)	79	134
	Marker (MRK)	276,903 (3.9%)	385	635	23,432 (0.7%)	47	212	300,335 (2.9%)	247	533
	Manhole cover (MAN)	26,104 (0.4%)	36	119	5186 (0.2%)	10	36	31,290 (0.3%)	25	95
	Curbing (CUR)	257,835 (3.7%)	358	394	4284 (0.1%)	8	67	262,119 (2.6%)	215	351
	Shoulder (SHO)	19,593 (0.3%)	27	136	3626 (0.1%)	7	35	23,219 (0.2%)	19	107
Backgrounds	Asphalt (ASP)	5,820,803 (82.6%)	8095	3791	0 (0.0%)	0	0	5,820,803 (57.2%)	4790	4933
	Concrete (CON)	0 (0.0%)	0	0	2,888,409 (92.2%)	5823	414	2,888,409 (28.4%)	2377	2874
All		7,049,605 (100%)	9,801	-	3,133,724 (100%)	6,313	-	10,183,329 (100%)	8,376	-

**Table 3:** Precisions, recalls, and F-scores of different classification models using UCF-PAVE 2017.

Metric	Method	Asphalt	Marker	Manhole	Patch	Pothole	Crack Seal	Shoulder	Curbing	Joint	Crack	Concrete	Avg
Precision	VGG16	0.941	0.91	0.828	0.66	0.547	0.501	0.623	0.92	0.733	0.709	0.963	0.758
	VGG19	0.938	0.917	0.878	0.765	0.587	0.474	0.571	0.916	0.713	0.726	0.96	0.768
	ResNet50	0.94	0.897	0.887	0.76	0.487	0.412	0.562	0.923	0.741	0.67	0.964	0.749
	DenseNet121	0.94	0.9	0.863	0.773	0.574	0.549	0.612	0.91	0.735	0.691	0.963	0.774
	M-VGG16	0.969	0.953	0.841	0.885	0.844	0.707	0.88	0.975	0.837	0.742	0.98	0.874
	M-VGG19	0.968	0.948	0.938	0.888	0.784	0.731	0.916	0.965	0.865	0.776	0.979	0.887
	M-ResNet50	0.962	0.957	0.93	0.908	0.825	0.704	0.911	0.963	0.8	0.756	0.981	0.881
	M-DenseNet121	0.967	0.947	0.911	0.874	0.818	0.823	0.926	0.965	0.829	0.733	0.98	0.889
	MCNN-EarlyFusion	0.966	0.945	0.924	0.882	0.842	0.788	0.91	0.974	0.845	0.786	0.98	0.895
	MCNN-MidFusion	0.982	0.948	0.944	0.945	0.788	**0.808**	**0.944**	0.978	0.867	0.812	0.983	0.909
	**A+MCNN (ours)**	**0.983**	**0.959**	**0.954**	**0.956**	**0.934**	0.789	0.929	**0.979**	**0.879**	**0.839**	**0.987**	**0.926**
Recall	VGG16	0.971	0.896	0.74	0.688	0.299	0.085	0.394	0.942	0.676	0.352	0.989	0.639
	VGG19	0.978	0.886	0.693	0.622	0.206	0.08	0.43	0.944	0.7	0.345	0.99	0.625
	ResNet50	0.975	0.902	0.677	0.618	0.368	0.139	0.524	0.938	0.676	0.379	0.986	0.653
	DenseNet121	0.976	0.898	0.709	0.615	0.305	0.092	0.466	0.948	0.684	0.376	0.988	0.642
	M-VGG16	0.979	0.947	0.959	0.884	0.812	0.693	0.903	0.965	0.808	0.611	0.989	0.868
	M-VGG19	0.981	0.953	0.941	0.898	0.884	0.64	0.895	0.969	0.785	0.586	0.991	0.866
	M-ResNet50	0.983	0.931	0.911	0.822	0.76	0.612	0.856	0.972	0.841	0.567	0.988	0.84
	M-DenseNet121	0.977	0.953	0.938	0.85	0.806	0.514	0.872	0.974	0.831	0.614	0.99	0.847
	MCNN-EarlyFusion	0.982	0.962	0.934	0.882	0.788	0.652	0.908	0.969	0.819	0.574	0.991	0.86
	MCNN-MidFusion	0.984	0.968	0.951	0.94	**0.907**	0.752	0.916	0.976	0.835	0.752	0.991	0.906
	**A+MCNN (ours)**	**0.987**	**0.969**	**0.953**	**0.943**	0.886	**0.788**	**0.944**	**0.986**	**0.842**	**0.761**	**0.991**	**0.914**
F-Score	VGG16	0.956	0.903	0.782	0.674	0.386	0.145	0.483	0.931	0.703	0.471	0.976	0.674
	VGG19	0.958	0.901	0.774	0.686	0.306	0.137	0.49	0.93	0.707	0.468	0.975	0.667
	ResNet50	0.957	0.9	0.768	0.682	0.419	0.207	0.542	0.93	0.707	0.484	0.975	0.688
	DenseNet121	0.958	0.899	0.778	0.685	0.399	0.158	0.53	0.928	0.709	0.487	0.975	0.682
	M-VGG16	0.974	0.95	0.896	0.884	0.827	0.7	0.891	0.97	0.822	0.67	0.984	0.87
	M-VGG19	0.974	0.95	0.939	0.893	0.831	0.682	0.906	0.967	0.823	0.667	0.985	0.874
	M-ResNet50	0.972	0.943	0.92	0.863	0.791	0.655	0.883	0.968	0.82	0.648	0.984	0.859
	M-DenseNet121	0.972	0.95	0.924	0.862	0.812	0.633	0.898	0.969	0.83	0.668	0.985	0.864
	MCNN-EarlyFusion	0.974	0.953	0.929	0.882	0.814	0.714	0.909	0.971	0.832	0.663	0.986	0.875
	MCNN-MidFusion	0.983	0.958	0.947	0.942	0.843	0.779	0.93	0.977	0.851	0.781	0.987	0.907
	**A+MCNN (ours)**	**0.985**	**0.964**	**0.953**	**0.949**	**0.909**	**0.788**	**0.936**	**0.982**	**0.860**	**0.798**	**0.989**	**0.920**

**Table 4:** Comparison of computational costs for different classification approaches.

Computational Costs	Single-Scale Baselines	Multi-Scale Baselines	MCNN-EarlyFusion	MCNN-MidFusion	A+MCNN
Number of parameters	51 M	51 M	62 M	88 M	95 M
Training time/epoch	107.9 s	110 s	96.6 s	181.7 s	199.4 s
Inference time/100 batches	4.0 s	4.2 s	5.5 s	8.0 s	8.7 s

**Table 5:** Classification results on UCF-PAVE 2017 dataset using the CNN presented in [31].

Metric	Method	Asphalt	Marker	Manhole	Patch	Pothole	Crack Seal	Shoulder	Curbing	Joint	Crack	Concrete	Avg
Precision	CNN	0.921	0.893	0.823	0.790	0.427	0.305	0.514	0.925	0.709	0.664	0.945	0.675
	M-CNN	0.942	0.918	0.881	0.773	0.576	0.572	0.740	0.939	0.734	0.648	0.955	0.756
Recall	CNN	0.980	0.853	0.407	0.457	0.076	0.052	0.249	0.907	0.610	0.241	0.976	0.491
	M-CNN	0.974	0.919	0.544	0.596	0.202	0.147	0.646	0.943	0.738	0.433	0.989	0.571
F-Score	CNN	0.950	0.873	0.545	0.579	0.128	0.089	0.335	0.916	0.656	0.354	0.960	0.527
	M-CNN	0.958	0.918	0.673	0.673	0.299	0.234	0.690	0.941	0.736	0.519	0.972	0.620

## Data Availability

The data presented in this study are available on request from the corresponding author. The data are not publicly available due to the legal considerations.
